# Update on the psychometric properties and minimal important difference (MID) thresholds of the FACT-M questionnaire for use in treatment-naïve and previously treated patients with metastatic Merkel cell carcinoma

**DOI:** 10.1186/s12955-020-01402-3

**Published:** 2020-05-19

**Authors:** Murtuza Bharmal, Sandra Nolte, Mickaël Henry-Szatkowski, Meliessa Hennessy, Michael Schlichting

**Affiliations:** 1 EMD Serono, Rockland, MA USA; 2 ICON plc, Munich, Germany; 3 Charité – Universitätsmedizin Berlin, corporate member of Freie Universität Berlin, Humboldt-Universität zu Berlin, and Berlin Institute of Health, Medical Department, Division of Psychosomatic Medicine, Berlin, Germany; 4 ICON plc, Lyon, France; 5 at the time of the study at EMD Serono, Billerica, MA USA; 6 Merck KGaA, Darmstadt, Germany

**Keywords:** Health-related quality of life, Merkel cell carcinoma, FACT-M questionnaire, Psychometrics, Validity and reliability, Patient reported outcome, Self report, Minimal important difference

## Abstract

**Objectives:**

For valid and reliable assessment of patients’ Health-Related Quality of Life (HRQoL), it is crucial to use psychometrically robust instruments. In the context of rare diseases such as Merkel cell carcinoma (MCC), validated disease-specific instruments are often not available. The Functional Assessment of Cancer Therapy – Melanoma (FACT-M) was originally developed for use in melanoma. Its psychometric performance for use in MCC and minimal important difference (MID) thresholds have been previously reported based on a cohort of metastatic MCC patients who had disease progression following one or more prior line of chemotherapy (NCT02155647 Part A; *n* = 70). Since then, new data from the phase II JAVELIN Merkel 200 trial among treatment-naïve patients are available (NCT02155647 Part B; *n* = 102). This study aims to increase accuracy and precision of previously established psychometric properties and MID thresholds of FACT-M in metastatic MCC patients.

**Methods:**

Published qualitative research suggests that patients with metastatic MCC had similar experiences and described similar concepts associated with their disease independent of whether they were treatment naïve or had prior treatment. Therefore, it was deemed appropriate to pool FACT-M data from Part A (previously treated) and Part B (treatment-naïve) cohorts for this study. Construct validity was assessed by evaluating item-factor correlations (convergent validity) and known-groups validity using ECOG performance status 0 versus 1. Concurrent validity was assessed using EQ-5D items. Internal consistency reliability was assessed using Cronbach’s α. Anchor- and distribution-based approaches were used to derive MID thresholds.

**Results:**

Overall, psychometric tests based on various validity (convergent, known-groups, concurrent) and reliability (Cronbach α) analyses confirmed previous findings in that FACT-M performs well in MCC patients. MID thresholds derived from this study are largely in line with previously established thresholds with some minor adjustments.

**Conclusions:**

In the context of rare diseases, which often have limited data available for psychometric testing, a reasonably large MCC patient sample was available for this study, enhancing accuracy and precision of previously established FACT-M psychometric properties and MID thresholds with only small deviations for use in metastatic MCC patients. Results suggest that the FACT-M is suitable for Merkel cell carcinoma regardless of patients’ treatment status.

**Trial registration:**

This study is a pre-planned post-hoc analysis conducted on data collected in Part A and Part B of the JAVELIN Merkel 200 trial. This trial was registered on 2 June 2014 with ClinicalTrials.gov as NCT02155647.

## Background

The importance of including the patient’s voice in clinical trials is well established [1, 2]. The most common approach to incorporate the patient perspective is the collection of patient-reported outcomes (PRO) data. An important prerequisite of obtaining high quality self-report data from the patient for valid inferences from these data [3] is the use of psychometrically robust PRO instruments. However, in the context of a rare disease such as Merkel cell carcinoma (MCC), disease-specific PRO instruments are often not available. As a result, PRO instruments have to be developed de novo or well-established PRO instruments have to be used from disease areas that are reasonably comparable to the disease of interest.

The phase II, single-arm JAVELIN Merkel 200 trial (NCT02155647) includes metastatic MCC patients who had disease progression following one or more prior line of chemotherapy (Part A) [4] or patients who were treatment naïve at study inclusion (Part B) [5]. As part of this trial, a range of PRO data was collected. In light of the lack of well-validated MCC-specific PRO instruments, the melanoma-specific Functional Assessment of Cancer Therapy – Melanoma (FACT-M) and EQ-5D-5 L questionnaires were used to assess patients’ self-reported health-related quality of life (HRQoL) while receiving avelumab. To ensure the suitability of the FACT-M for use in MCC, it is crucial to test its psychometric performance in this patient population. A first publication exploring the psychometric performance of the FACT-M in MCC provided evidence for the suitability of the FACT-M for use in MCC patients [6]. These analyses had been based on patients who had already received second-line or later treatment (Part A). Since the publication of these first results, new PRO data obtained from treatment-naïve patients (Part B) became available. As the suitability of the FACT-M for use in MCC needs to be established for both MCC patient groups, it is crucial to repeat the psychometric analyses on Part B patients. For this, it was deemed justified and advantageous to pool the two samples for several reasons. First, the combined sample size is substantially larger than the individual samples ensuring more sensitive analyses and robustness of the results [7]. Second, qualitative interviews with patients from both study parts indicated similar experiences related to their MCC diagnosis and its management, and regarding perceived benefits and clinical changes experienced during the trial [8, 9]. Third, it is crucial to establish that the FACT-M is suitable for the application in MCC in general, irrespective of treatment status at study inclusion. By including a greater range of MCC patients by pooling the two samples, validity evidence can be extended to a more heterogeneous MCC patient population. Finally, for the definition of minimal important difference (MID) thresholds, it is important to establish thresholds that can be applied to the entire MCC population. This warrants comparability of results obtained from different patient groups.

Hence, this study aims at confirming previously reported psychometric properties and MID thresholds of the FACT-M [6] in patients with MCC. By using pooled Part A and B trial data, the sample size could be increased substantially compared to the previous publication [6], enhancing accuracy and precision of psychometric tests and MID thresholds. This new set of analyses is intended to complement/replace the Part A results by providing a more robust piece of evidence applicable to a broader patient population consisting of previously treated (Part A) and treatment-naïve (Part B) MCC patients.

## Methods

### Study design

The JAVELIN Merkel 200 trial is a single-arm, open-label, multi-center, international phase II study consisting of two parts. For inclusion in either of the two parts, eligible patients had histologically confirmed metastatic MCC (stage IV), were at least 18 years of age, and had an Eastern Cooperative Oncology Group (ECOG) performance score of 0 or 1. Patients were excluded if they had autoimmune or various other conditions [4, 5]. For inclusion in the first part (Part A), patients had already received and failed one line or more of chemotherapy treatment for metastatic MCC. The planned sample size for Part A was 84 patients, giving the study 87% power to assess clinical activity [4]. For inclusion in the second part (Part B), patients had to be treatment naïve to systemic therapy [5]. Further details of the study design as relevant to the present study are reported elsewhere [6].

### Study population

For the purpose of substantiating previously reported psychometric performance and MID thresholds of the FACT-M [6], the intention-to-treat trial populations of Part A (*n* = 88) and B (*n* = 116) were pooled, leading to a combined sample size of *n* = 204. As not all patients provided baseline data, a PRO analysis set (PAS) was defined consisting of *n* = 172 patients (Part A: *n* = 70; Part B: *n* = 102). To assess the ability of the FACT-M to detect change and derive MID thresholds, these analyses are based on data collected at week 7 (*n* = 121). Week 7 was chosen as the most suitable time point to measure responsiveness of the FACT-M, as the main tumor response is expected at that time. The pooled sample is based on respective Part A/B data cut-off date 14 September 2018.

### Patient-reported outcome assessments

The FACT-M and EQ-5D instruments were used to capture PRO data in the JAVELIN Merkel 200 trial.

The FACT-M questionnaire includes 51 items grouped into nine scores, including six subscale and three summary scores [10, 11]. Three additional MCC-specific FACT-M scores have been established previously for use in MCC [6]. The recall period of all FACT-M items is 7 days and items are scored on a 5-point scale, ranging from 0 = ‘not at all’ to 4 = ‘very much’. For all subscale, summary and MCC-specific FACT-M scores, a higher score indicates higher well-being. For the purpose of this study, the psychometric properties of the FACT-M and its various subscale and summary scores, including the MCC-specific FACT-M scores [6], are documented. The latter include the MCC-specific subscale Physical Function (PF; six FACT-M items), Psychological Impact (PI; six FACT-M items) and the MCC summary score (PF + PI). While the previous publication established and tested the psychometric performance of the newly defined MCC-specific scores on a subset of MCC patients [6], the present study aims to substantiate the psychometric properties but also establish MID thresholds for PF, PI and MCC summary score.

The EQ-5D-5 L questionnaire includes five single-item dimensions (i.e., mobility, self-care, usual activities, pain/discomfort, anxiety/depression) with five response levels each (5 L), and a vertical visual analogue scale (VAS, i.e. EQ VAS). There is no recall period in the EQ-5D items, i.e., the items ask patients to assess their health status on that particular day of filling out the questionnaire. For both the EQ VAS and the EQ-5D index score, a higher score indicates better health status, and a positive change reflects an improvement [12].

### Statistical analysis

Sociodemographic and clinical characteristics are described (mean, median and range for quantitative variables; percentages for qualitative variables) and compared across study Part A and Part B (t-tests for continuous variables; Chi-square tests for categorical variables).

For the confirmation of the psychometric properties of the FACT-M in the MCC population, previous analyses based on the Part A sample [6] were largely repeated using pooled data. First, using baseline data, internal consistency of all FACT-M scales was explored using Cronbach’s alpha [13]. In addition, and new to the analyses of the pooled data presented herein, McDonald’s (1999) [14] coefficient omega was calculated as an alternative to alpha to assess the respective reliability of the six FACT-M subscales and the two MCC-specific subscales. It is calculated as the ratio of the common (i.e., true-score) variance to the total variance (i.e., common plus error variance). Omega has been shown to overcome deficiencies of alpha and has been strongly recommended as a more robust estimate of reliability compared with alpha [15, 16]. In this article, omega is based on one-factor models [16] applying confirmatory factor analysis (CFA) and calculated for the six FACT-M subscales and the two MCC-specific subscales, respectively.

For construct validity, baseline data were used to test for item convergent and divergent validity, i.e., multi-scaling analyses to test item-to-scale correlations (r) where individual items are expected to correlate highly with their own domain (r ≥ 0.4; convergent validity) and correlate higher with their own compared to correlations with other domains (divergent validity). To substantiate the construct validity of both the FACT-M and the newly developed two MCC-specific subscales for use in MCC, CFAs were carried out. In addition to a six-factor FACT-M model, a four-factor model was specified containing the four FACT-G subscales and a two-factor model containing the melanoma subscale and the melanoma surgery scale. The two MCC-specific subscales were run as a separate two-factor model. Clinical validity was assessed using subgroups defined by ECOG performance status (PS) 0 (=fully active) versus 1 (=restricted in physically strenuous activity). Criterion (concurrent) validity was assessed using the EQ VAS and EQ-5D index score and adding the 5 EQ-5D single items which had not been tested in the previous publication [6]. The ability of the FACT-M to detect change over time was based on variable ‘change in tumor size’ to assess group differences, comparing baseline with data assessed at week 7. These analyses were repeated on EQ VAS to explore group differences across categories ‘improved’, ‘stable’ and ‘worsened’. The latter analysis had not been tested in the previous publication [6] but was deemed an important addition given that a patient-reported variable, such as the EQ VAS, was expected to categorize patients into more patient-relevant groups compared with variable ‘change in tumor size’. Change is expressed as means as well as effect size.

Closely following the Part A analyses [6], MID thresholds to define ‘meaningful improvement’ and ‘meaningful worsening’ on the FACT-M were derived from the pooled week-7 data. Thresholds were calculated for each FACT-M score using a combination of anchor- and distribution-based methods, a common approach to derive responder definitions and minimally important difference thresholds [17–19]. For the anchor-based approach, the initial analyses using Part A data applied variable ‘change in tumor size’ as an anchor [6]. Similar to the rationale behind FACT-M responsiveness analyses however, it was decided to again use a patient-reported anchor [20], i.e., ‘change in EQ VAS’, as the preferred anchor (MID = 7 points) given the higher correlation between FACT-M and EQ VAS compared with weaker correlations between FACT-M and ‘change in tumor size’. Correlations of reasonable size between target instrument and anchor are a prerequisite for being a suitable anchor [21]. Following recommended anchor selection criteria, a PRO instrument is also the anchor of choice [22]. The remaining methods for MID definition replicated the already published analyses on Part A data [6].

## Results

### Study population

Baseline socio-demographic and clinical characteristics of patients included in the PAS of the FACT-M (*n* = 172) are presented in Table 1. A majority of patients were male (70.3%) with a mean age of 71.6 years (SD = 10.4). The median time since diagnosis was 2 years. The ECOG score indicated that over half of the total population (60.5%) were fully active (ECOG PS = 0), while the remaining 39.5% were restricted in physically strenuous activity (ECOG PS = 1). Mean baseline tumor size was 88.6 mm.

**Table 1:** Sociodemographic / clinical characteristics of Part A and Part B patients of JAVELIN Merkel 200 trial, FACT-M PRO analysis set (*n* = 172)*

Sociodemographic / clinical characteristics	Study part			p-value**
	Part A (n = 70)	Part B (n = 102)	Total (n = 172)	
Gender n (%)				
Male	52 (74.3%)	69 (67.6%)	121 (70.3%)	0.3490
Female	18 (25.7%)	33 (32.4%)	51 (29.7%)	
Age (years)				
Mean (SD)	70.20 (11.19)	72.53 (9.81)	71.58 (10.42)	0.1505
Median	73.00	73.50	73.00	
Range	33.00–88.00	45.00–93.00	33.00–93.00	
Pooled geographic region n (%)				
North America	40 (57.1%)	27 (26.5%)	67 (39.0%)	0.0001
Europe	22 (31.4%)	63 (61.8%)	85 (49.4%)	
Rest of the World	8 (11.4%)	12 (11.8%)	20 (11.6%)	
ECOG PS at baseline, n (%)				
ECOG PS 0	38 (54.3%)	66 (64.7%)	104 (60.5%)	0.1697
ECOG PS 1	32 (45.7%)	36 (35.3%)	68 (39.5%)	
Site of primary tumor, n (%)				
Non-skin	9 (12.9%)	7 (6.9%)	16 (9.3%)	0.1097
Skin	55 (78.6%)	91 (89.2%)	146 (84.9%)	
Not applicable	0 (0.0%)	4 (3.9%)	4 (2.3%)	
Missing	6 (8.6%)	0 (0.0%)	6 (3.5%)	
Tumor size at baseline (mm)				
n (missing)	61 (9)	100 (2)	161 (11)	
Mean (SD)	103.69 (79.68)	79.46 (58.48)	88.64 (68.09)	0.0280
Median	83.00	64.00	66.00	
Range	16.00–404.00	0.00–288.00	0.00–404.00	
Time since initial diagnosis (years)				
Mean (SD)	2.19 (0.80)	2.27 (0.76)	2.24 (0.78)	0.4631
Median	2.00	2.00	2.00	
Min - Max	1.00–3.00	1.00–3.00	1.00–3.00	
Time since first metastatic disease (months)				
Mean (SD)	16.94 (23.44)	5.46 (7.66)	10.13 (16.98)	<.0001
Median	9.49	2.33	5.73	
Min - Max	1.51–156.75	0.36–49.58	0.36–156.75	

ECOG PS = Eastern Cooperative Oncology Group Performance Status (0 = fully active; 1 = restricted in physically strenuous activity); SD = standard deviation;

*Of note, in the process of data cleaning of a subsequent Part B data cut, baseline data of four patients were set to missing as their assessment had taken place after treatment initiation. For the present analysis, however, it was deemed irrelevant that treatment had already started, as the psychometric properties of the FACT-M should not be influenced by assessment time point. Hence, these patients are included in the psychometric analyses, explaining discrepancies between the sample size reported herein and the sample size used for the analyses of HRQoL outcomes over time where *n* = 98 patients (instead of n = 102) are included in PAS of the FACT-M

** P-value for between-group comparisons: T-test for continuous variables, Chi^2^ or Fisher’s exact for categorical variables

When comparing Part A and Part B samples, some differences were apparent. Mean tumor size was larger for Part A compared with Part B patients (103.7 mm [SD = 79.7] versus 79.5 mm [SD = 58.5]) and the median time since patients reached first metastatic disease was 9.5 months for Part A and 2.3 months for Part B, respectively (5.7 months for Part A and B pooled).

### Internal consistency reliability

As shown in Table 2, Cronbach’s alpha coefficients were all superior to the recommended threshold of 0.7 supporting the internal consistency of all FACT-M generated scores. The estimates of coefficient omega were either identical to or slightly above alpha for all eight subscales, ranging between 0.80 and 0.89.

**Table 2:** Internal consistency reliability and construct (convergent/divergent, clinical) validity of the FACT-M in the MCC population (*n* = 172)

FACT-M subscale, summary and MCC-specific scores	# items	Cronbach’s alpha^[a]^	McDonald’s coefficient omega	Scale structure		ECOG PS (mean [SD])		
				Range of item-subscale correlations^[b]^	Convergent^[c]^/ divergent^[d]^ validity (% of items)	0 (*n* = 106)	1 (*N* = 66)	p-value^[e]^
FACT-G subscales								
Physical well-being	7	0.84	0.85	0.53–0.71	100 / 57	24.0 (4.2)	21.3 (5.6)	**<.001**
Social/Family well-being^[f]^	6	0.83	0.85	0.55–0.70	100 / 100	22.5 (5.4)	21.6 (5.7)	0.308
Emotional well-being	6	0.80	0.80	0.36–0.70	83 / 67	18.1 (4.1)	16.8 (5.0)	**0.0499**
Functional well-being	7	0.89	0.89	0.52–0.77	100 / 71	18.1 (6.7)	15.8 (6.4)	**0.028**
Melanoma-specific subscales								
Melanoma subscale	16	0.84	0.86	0.19–0.76	50 / 38	54.3 (7.5)	50.6 (9.0)	**0.004**
Melanoma surgery scale	8	0.82	0.83	0.20–0.77	75 / 75	27.0 (5.3)	24.4 (7.1)	**0.007**
Summary scales								
FACT-M TOI	30	0.94	–	–	–	96.4 (16.7)	88.1 (19.5)	**0.003**
FACT-G Total score	27	0.93	–	–	–	82.8 (16.2)	75.5 (18.1)	**0.007**
FACT-M Total score	43	0.95	–	–	–	137.0 (22.3)	126.6 (25.8)	**0.006**
MCC-Specific scores								
Physical function score (PF)	6	0.89	0.89	0.61–0.76	100 / 100	17.2 (5.5)	14.3 (5.9)	**0.001**
Psychological impact score (PI)	6	0.84	0.85	0.55–0.72	100 / 100	18.4 (4.3)	17.0 (5.2)	0.064
MCC summary score	12	0.90	–	–	–	35.5 (8.7)	31.3 (10.0)	**0.004**

# = number; ECOG PS = Eastern Cooperative Oncology Group performance score (0 = fully active; 1 = restricted in physically strenuous activity); MCC = Merkel cell carcinoma; SD = Standard deviation, TOI = Trial Outcome Index

[a] Recommended threshold α > 0.7; [b] Pearson correlation coefficients; [c] % of items correlated with its own dimension ≥0.4; [d] % of items correlated with its own dimension higher that the correlation with any other dimension; [e] p-value from t-test of score between the two ECOG performance status groups at baseline; [f] convergent and divergent validity were calculated without the ‘satisfaction with sex life’ item due to high item missingness resulting from response to this item being voluntary

### Convergent and divergent validity using multi-trait analysis

Multi-trait analysis, which is based on inter-item correlations, requires all items to be non-missing. As soon as one item is missing the patient is excluded from the analysis. Optional item GS7 as part of the Social well-being scale asks patients about their satisfaction with sex life. As this item exhibited high missingness, multi-trait analysis was conducted twice: Once with all FACT-M items (51 items), once excluding item GS7 (50 items). Exclusion of GS7 doubled the sample size available for these analyses. As the two sets of analyses led to similar results, results based on 50 items are presented Table 2, as this analysis provided a more robust sample size.

Convergent validity was generally good with 100% of items meeting the item-convergent validity criterion for three of the four FACT-G subscales and 83% for Emotional well-being. The two melanoma-specific subscales showed lower levels of correlations, i.e. 50 and 75%, respectively. The percentage of items that met the divergent validity criterion was highest for Social well-being, with all items (100%) meeting the divergent validity criterion, and lowest in the Melanoma subscale, with 38% of items meeting the divergent validity criterion.

Multi-trait analysis of the scaling structure of the two proposed MCC-specific FACT-M subscales (PF and PI) involving six items each was tested in a simple model using the selected 12 items only. Results indicated perfect item convergent and divergent validity (Table 2).

### Construct validity using confirmatory factor analysis

We ran into model conversion issues when specifying the six-factor FACT-M model, which was likely a combination of the sample size which was rather small for CFA and the size of the model (six factors with 51 items or 50 items when taking out item GS7, respectively).

In contrast, the four-factor FACT-G model converged (based on *n* = 170, excluding item GS7) with overall satisfactory fit indices, with a root mean square error of approximation (RMSEA) of 0.077 (90% confidence interval [CI], 0.068–0.086), a standardized root mean square residual (SRMR) of 0.076 and a comparative fit index (CFI) of 0.874. All but one factor loading of the Emotional well-being subscale were at least 0.5 or higher, with most being well above 0.6. The two-factor model containing the melanoma subscale and the melanoma surgery scale converged as well, with fit indices suggesting a worse model fit compared with the four-factor model, with RMSEA of 0.094 (90% CI, 0.085–0.103), SRMR of 0.083 and CFI of 0.773. Especially the factor loadings of the melanoma subscale showed some very small factor loadings, with five being below 0.4 and two being below 0.3. Finally, the two MCC-specific subscales showed excellent model fit, with RMSEA of 0.072 (90% CI, 0.049–0.094), SRMR of 0.057 and CFI of 0.957. All factor loadings were above 0.6. Of note, none of these models allowed for any correlated errors or other model adjustments.

### Clinical/known groups validity

As shown in Table 2, mean FACT-M scores were larger for the group of patients who were fully active (ECOG PS = 0) compared to those restricted in physically strenuous activity (ECOG PS = 1) across all but two FACT-M scores, reflecting the better functioning of the former group. The difference between the two groups led to *p*-values below 0.05 for all scores except for Social well-being and the MCC-specific PI score.

### Concurrent validity

As shown in Table 3, correlation coefficients between FACT-M subscale, summary and MCC-specific scores and EQ-5D items were generally medium to large (i.e., > 0.4), except for FACT-M Social well-being which showed small correlations at best. Apart from this subscale, all other FACT-M scores were also highly correlated with the two summary EQ-5D scores (VAS and Index). Finally, correlations between FACT-M scores and EQ-5D items were highest in absolute value for scores assessing similar or associated concepts and lower for those measuring different concept, e.g. Emotional well-being correlating highest with EQ-5D anxiety/depression.

**Table 3:** Pearson correlation coefficients between FACT-M and EQ-5D scores at baseline (n = 172)*

Score at baseline	EQ-5D mobility	EQ-5D self-care	EQ-5D usual activity	EQ-5D pain/ discomfort	EQ-5D anxiety/ depression	EQ VAS	EQ-5D Index
FACT-G subscales							
Physical well-being	− 0.43**	− 0.39	− 0.64	− 0.68	− 0.43	0.59	0.65
Social/Family well-being	−0.21	− 0.14	− 0.19	− 0.12	− 0.24	0.22	0.22
Emotional well-being	− 0.31	− 0.27	− 0.45	−0.41	− 0.59	0.52	0.48
Functional well-being	−0.46	−0.33	− 0.63	−0.51	− 0.52	0.56	0.61
Melanoma-specific subscales							
Melanoma subscale*	−0.49	−0.41	− 0.70	−0.65	− 0.49	0.63	0.68
Melanoma surgery scale	−0.38	−0.42	− 0.53	−0.45	− 0.26	0.45	0.50
Summary scales							
FACT-M TOI	−0.50	−0.41	− 0.72	−0.66	− 0.53	0.65	0.71
FACT-G Total score	−0.45	−0.35	− 0.60	−0.54	− 0.55	0.59	0.62
FACT-M Total score	−0.49	−0.39	− 0.67	−0.60	− 0.56	0.64	0.67
MCC-specific scores							
Physical function (PF)	−0.56	−0.40	− 0.73	−0.59	− 0.47	0.57	0.68
Psychological impact (PI)	−0.30	−0.26	− 0.45	−0.43	− 0.58	0.50	0.48
MCC summary score	−0.49	−0.38	− 0.67	−0.57	− 0.57	0.60	0.66

* Negative correlations are due to the different scoring of the two instruments, with high EQ-5D item scores reflecting greater health/function problems, whereas for scores from the FACT-M, higher scores indicate better well-being or health status

** Grey shadings indicate correlations between EQ-5D item / index / EQ VAS scores expected to correlate highest with FACT-M subscale / summary / MCC-specific scores

TOI = Trial Outcome Index; MCC = Merkel cell carcinoma; *n = 171 for Melanoma subscale

### Ability of the FACT-M scores to detect change over time

To explore the ability of the FACT-M scores to detect change over time ‘change in tumor size’ was used as an anchor replicating the psychometric analyses carried out on Part A data [6]. As information on the percentage change in tumor size was only available for 105 patients, this first set of analysis is based on a sample size of *n* = 105. The FACT-M showed a general logical pattern of improvement in FACT-M scores for the improved group (except for Physical well-being and PF which were negative but close to 0), worsening in FACT-M scores for the worsened group (except for Emotional well-being and PI which were positive but of small amplitude) and score changes generally negative but close to zero for the stable group (except for Emotional well-being and PI). The main departure from a clearly monotonous pattern was seen in the two melanoma-specific subscales where the stable group indicated the largest decrease (worsening) in scores.

When using the EQ VAS as an additional anchor to differentiate between groups of change (*n* = 121), again a logical pattern of FACT-M scores was observed. That is, improvement was observed for the improved group, worsening in FACT-M scores for the worsened group and score changes generally negative (except for Emotional well-being) but close to 0 for the stable group. The main departure from a clearly monotonous pattern was seen in the Social well-being scale where the stable group indicated the largest decrease (worsening) in scores. The same patterns were observed when expressing change in form of effect sizes for the different groups (Table 4).

**Table 4:** Ability to detect change of the FACT-M in the MCC population by comparing change in score from baseline to week 7 across change groups defined by percentage change in tumor size (n = 105) and change in EQ VAS (*n* = 121), respectively

FACT-M subscale, summary and MCC-specific scores	Percent change in tumor size; mean (effect size)				Change in EQ VAS (MID = 7); mean (effect size)			
	Reduction ≤ − 30%^[a]^ (*N* = 47)	Reduction > − 30% to ≤0 (*N* = 14)	Increase > 0% (*N* = 44)	p-value^[b]^	Improved ≥7 points (*n* = 35)	Stable > − 7 to < 7 (*n* = 56)	Worsened ≤ − 7 points (*n* = 30)	p-value^[b]^
FACT-G subscales								
Physical well-being	−0.02 (− 0.01)	−0.50 (− 0.11)	− 1.86 (− 0.36)	0.101	0.46 (0.10)	−0.54 (− 0.11)	−3.17 (− 1.03)	**< 0.001**
Social/Family well-being	0.47 (0.09)	− 0.21 (− 0.06)	− 1.55 (− 0.24)	0.209	0.72 (0.15)	− 1.19 (− 0.21)	− 0.13 (− 0.02)	0.272
Emotional well-being	1.38 (0.35)	0.79 (0.14)	0.23 (0.05)	0.292	2.37 (0.63)	0.99 (0.20)	−2.03 (− 0.65)	**<.0001**
Functional well-being	0.06 (0.01)	− 0.43 (− 0.06)	− 2.16 (− 0.30)	0.164	0.34 (0.05)	− 0.91 (− 0.12)	−2.24 (− 0.40)	0.165
Melanoma-specific subscales								
Melanoma subscale	1.64 (0.21)	−2.64 (− 0.44)	− 2.32 (− 0.27)	**0.006**	1.74 (0.24)	− 0.18 (− 0.02)	−4.63 (− 0.69)	**<.0001**
Melanoma surgery scale	1.50 (0.36)	−1.79 (− 0.48)	−1.16 (− 0.15)	**0.043**	0.69 (0.12)	0.02 (0.02)	− 0.67 (− 0.09)	0.619
Summary scales								
FACT-M TOI	1.68 (0.10)	− 3.57 (− 0.23)	− 6.34 (− 0.32)	**0.012**	2.54 (0.16)	− 1.63 (− 0.08)	−10.04 (− 0.76)	**< 0.001**
FACT-G Total score	1.89 (0.13)	− 0.36 (− 0.02)	− 5.34 (− 0.29)	**0.026**	3.90 (0.26)	− 1.65 (− 0.09)	−7.56 (− 0.56)	**0.001**
FACT-M Total score	3.53 (0.17)	−3.00 (− 0.14)	− 7.65 (− 0.30)	**0.009**	5.64 (0.27)	−1.83 (− 0.07)	−12.20 (− 0.67)	**<.0001**
MCC-Specific scores								
Physical function score (PF)	−0.30 (− 0.06)	−1.64 (− 0.32)	−2.18 (− 0.33)	0.100	−0.09 (− 0.01)	−0.95 (− 0.15)	−3.60 (− 0.79)	**0.002**
Psychological impact score (PI)	1.40 (0.32)	1.21 (0.22)	0.30 (0.07)	0.393	2.94 (0.71)	0.98 (0.20)	−2.10 (−0.62)	**<.0001**
MCC summary score	1.11 (0.14)	−0.43 (− 0.04)	−1.89 (− 0.19)	0.129	2.86 (0.33)	0.04 (0.00)	−5.70 (− 0.91)	**<.0001**

MCC = Merkel cell carcinoma; MID = Minimal important difference; TOI = Trial Outcome Index

[a] reduction in tumor size ≥30% was used as one anchor for MID thresholds; [b] *p*-value from ANOVA comparing score change from baseline to week 7 between the three groups based on change in tumor size or change in EQ VAS respectively

### MID thresholds

The minimum and maximum MID thresholds for the various FACT-M subscale and summary scores were derived using a combination of anchor- and distribution-based approaches. As shown in Table 5, results were largely in line with those derived from Part A data [6]. For Functional well-being, Melanoma surgery scale and FACT-M Trial Outcome Index (TOI) the minimum threshold was smaller when derived from the pooled data (each by one point), whereas for FACT-G Total score, the maximum threshold was larger by one point compared with the threshold derived from Part A data. All remaining thresholds were identical to those reported previously [6].

**Table 5:** Minimum and maximum MID thresholds derived from anchor- and distribution-based methods for FACT-M in MCC

FACT-M subscale and summary scores	0.2*SD_BL_	0.5*SD_BL_	SEM^[a]^	Anchor^[b]^	Range (min-max)^[c]^	Range rounded (min-max)^[d]^	Published MIDs for FACT-M^[e]^
FACT-G subscales							
Physical well-being	0.99	2.47	1.95	0.46	0.46–2.47	1–3	2–3 †
Social/Family well-being	1.09	2.74	2.26	0.72	0.72–2.74	1–3	
Emotional well-being	0.90	2.24	2.02	2.37	0.90–2.37	1–3	2†
Functional well-being	1.33	3.33	2.25	0.34	0.34–3.33	1–4	2–3†
Melanoma-specific subscales							
Melanoma subscale	1.65	4.13	3.31	1.74	1.65–4.13	2–5	2–4 ¥
Melanoma surgery scale	1.23	3.08	2.63	0.69	0.69–3.08	1–3	1–2 ¥
Summary scales							
FACT-M Trial Outcome Index (TOI)	3.40	8.50	4.30	2.54	2.54–8.50	3–9	5–9 ¥
FACT-G Total score	3.44	8.60	4.59	3.90	3.44–8.60	4–9	5–7 †
FACT-M Total score	4.83	12.08	5.61	5.64	4.83–12.08	5–12	
MCC-Specific scores							
FACT-M Physical function score	1.15	2.88	1.93	−0.09	1.15–2.88	2–3	
FACT-M Psychological impact score	0.94	2.35	1.90	2.94	0.94–2.94	1–3	
FACT-M MCC summary score	1.88	4.71	2.95	2.86	1.88–4.71	2–5	

[a] SEM is calculated using Cronbach’s alpha based on baseline scores; [b] The anchor used is the EQ VAS score with threshold MID = 7; [c] The range was derived from the minimum and maximum resulting from the various methods, with the exception of the MCC-specific scores where the smallest positive value was used as the negative value was implausible given that the thresholds are proposed to be used for both improvement and worsening; [d] While rounding was generally to the next higher integer, we rounded down in case the first decimal point was a zero; [e] FACT-M MIDs for melanoma published by: †Cella et al. (2002) [1]; ¥ Askew et al. (2009) [2]

## Discussion and conclusion

### Discussion

The main objective of this study was to confirm the psychometric properties of the FACT-M and MID thresholds for use in MCC patients, which had been previously obtained from Part A of the trial [6]. By using pooled Part A and B trial data, the sample size could be increased substantially compared to the previous publication [6], enhancing accuracy and precision of psychometric tests and MID thresholds as well as extending the applicability of the results to a broader patient population consisting of previously treated (Part A) and treatment-naïve (Part B) MCC patients. In addition to the FACT-M scores, preceding analyses on Part A data had resulted in the development of three additional scores to capture concepts most relevant to MCC patients. Item selection to generate these MCC-specific scores had taken into account results from psychometric analyses [6] and qualitative research to ensure that key concepts elicited from MCC patients were included [8, 9]. As these MCC-specific scores are new, the present study was also aimed at generating additional validity evidence and derive MID thresholds for these scores.

Results from the psychometric analyses of the present study are generally supportive of the construct validity of the FACT-M in MCC patients. Particularly, the MCC-specific scores showed strong psychometric properties as part of the multi-trait analysis as well as the CFA, suggesting that these subscales may be particularly suitable for this patient population. In contrast, the Melanoma subscale showed low item convergent and divergent validity as part of the multi-trait analysis. On closer inspection, these results seem to reflect that this subscale includes disease-specific symptoms not necessarily associated to each other (e.g., changes in skin, fevers, shortness of breath, headaches, aches and pains in bones, blood in stools), which are also partly specific to melanoma. Further, this subscale covers aspects related to physical, emotional or functional well-being, leading to higher correlations with these subscales. Therefore, suboptimal psychometric properties would be expected. The suboptimal performance of the melanoma subscale, in combination with the melanoma surgery scale, was further confirmed as part of the CFA. Even when taking out several poor-performing items from these subscales, as proposed by Swartz et al. (2012) [23], model fit could not be improved substantially, with RMSEA being slightly worse and SRMR and CFI slightly better compared with the two-factor model containing all original items of the two scales. Following from these analyses, both the FACT-G and the MCC-specific scores seem to be performing strongest in MCC and should be the focus when interpreting HRQoL of MCC patients, while both the melanoma subscale and melanoma surgery scale should be used with caution. In the context of CFA, however, we want to stress that these analyses may not be robust given the rather small sample size to carry out CFA. Therefore, replication of CFA in a larger sample of MCC patients is highly recommended.

In addition to construct validity, strong clinical validity evidence of the FACT-M was found, with patients who were fully active (ECOG PS = 0) showing higher scores compared to those who were restricted in physically strenuous activity (ECOG PS = 1). Correlations between FACT-M and EQ-5D scores were as expected, highest in absolute value for scores assessing similar or associated concepts, supporting the concurrent validity of the FACT-M.

The ability of the FACT-M to detect change was demonstrated, specifically when applying change in EQ VAS. Results of the analyses conducted with the variable ‘change in tumor size’ were also supportive but less conclusive than those using the EQ VAS, providing support for the choice of a patient-reported anchor, as also recommended in the literature [22]. Finally, the internal consistency reliability of all FACT-M scores was supported by Cronbach’s alpha values > 0.7. In summary, this study provides strong support for the suitability of the FACT-M for use in MCC patients.

Finally, the derived MIDs were generally consistent with those obtained in the preliminary analyses conducted on Part A data [6], although slight variations were seen. We recommend using the newly derived thresholds for interpreting change in MCC as measured by the FACT-M.

## Conclusion

The FACT-M was originally developed for use in melanoma. To justify its use in MCC, it was important to demonstrate satisfactory psychometric performance of the FACT-M when used in Merkel cell carcinoma patients. Results from qualitative research work support the FACT-M content validity, while the present quantitative analyses support its reliability, validity and ability to detect change in MCC patients. Therefore, the application of the FACT-M in MCC is deemed appropriate. While all FACT-M scores may be used, a shorter version of 12 items – the MCC-specific scores – may be considered, as these presented the strongest psychometric properties of the FACT-M in MCC. Finally, the MID thresholds established as part of this study can serve as a guide for interpreting change scores in other research and trials in Merkel cell carcinoma.

## Data Availability

The datasets generated and/or analyzed during the current study are not publicly available as the clinical trial is still on-going but are available from the corresponding author on reasonable request.

## Abbreviations

ECOG PS: Eastern Cooperative Oncology Group performance status
EQ-5D: EuroQol 5 dimensions – 5 levels
FACT-G: Functional Assessment of Cancer Therapy – General
FACT-M: Functional Assessment of Cancer Therapy – Melanoma
HRQoL: Health-related quality of life
MCC: Merkel cell carcinoma
MID: Minimally important difference
PF: Physical Function
PI: Psychological Impact
PRO: Patient reported outcome
SD: Standard deviation
SEM: Standard error of measurement
TOI: Trial Outcome Index
VAS: Visual analogue score
